# A study of motor imagery EEG classification based on feature fusion and attentional mechanisms

**DOI:** 10.3389/fnhum.2025.1611229

**Published:** 2025-07-16

**Authors:** Tingting Zhu, Hailin Tang, Lei Jiang, Yijia Li, Shijun Li, Zhijian Wu

**Affiliations:** ^1^School of Big Data and Computing, Guangdong Baiyun University, Guangzhou, China; ^2^Dropbox Inc., San Francisco, CA, United States; ^3^School of Computer Science, Wuhan University, Wuhan, China

**Keywords:** brain-computer interface, motor imagery, EEG, attention mechanism, feature fusion

## Abstract

**Introduction:**

Motor imagery EEG-based action recognition is an emerging field arising from the intersection of brain science and information science, which has promising applications in the fields of neurorehabilitation and human-computer collaboration. However, existing methods face challenges including the low signal-to-noise ratio of EEG signals, inter-subject variability, and model overfitting.

**Methods:**

We propose HA-FuseNet, an end-to-end motor imagery action classification network. This model integrates feature fusion and attention mechanisms to classify left hand, right hand, foot, and tongue movements. Its innovations include: (1) multi-scale dense connectivity, (2) hybrid attention mechanism, (3) global self-attention module, and (4) lightweight design for reduced computational overhead.

**Results:**

On BCI Competition IV Dataset 2A, HA-FuseNet achieved 77.89% average within-subject accuracy (8.42% higher than EEGNet) and 68.53% cross-subject accuracy.

**Conclusion:**

The model demonstrates robustness to spatial resolution variations and individual differences, effectively mitigating key challenges in motor imagery EEG classification.

## 1 Introduction

In recent years, Brain-Computer Interface (BCI), an emerging field at the intersection of brain science and information science, aims to establish direct communication and control channels between the brain and external devices, enabling bidirectional signal transmission (Jorgenson et al., 2015). Among BCI paradigms, Motor Imagery (MI) based on non-invasive Electroencephalography (EEG) has emerged as an important research direction owing to its low cost, high temporal resolution, and portability, with broad application prospects in areas such as motor function rehabilitation, intelligent human-computer interaction, and cognitive science research (Naddaf, 2023).

Motor imagery is a psychological process in which an individual activates physiological phenomena in the relevant brain regions in the brain by imagining a specific action in the absence of an actual action, thus providing the possibility of motor function compensation and device control. However, EEG signals face many challenges in motor imagery classification tasks due to their low signal-to-noise ratio, non-stationarity, and individual variability (Notice of the Ministry of Science and Technology of the People's Republic of China, 2021). Traditional methods rely on *a priori* knowledge in the field of neuroscience for manual feature design, and although the introduction of deep learning techniques has made some progress in automated feature extraction, there is still room for existing methods to improve classification accuracy and generalization due to the small size of the EEG dataset, the poor quality of the data, and the high real-time requirements (Poo et al., 2016).

Current EEG-based motor imagery classification methods face several key challenges: (1) Feature extraction often relies heavily on domain-specific neuroscience expertise, requiring strict frequency band filtering and high spatio-temporal resolution. This limits their adaptability for practical home-based or personalized applications; (2) The inherent low signal-to-noise ratio (SNR) of EEG signals and significant inter-subject variability result in unstable model performance across individuals, limiting classification accuracy to below-desired levels; (3) EEG datasets are typically limited in size, whereas deep learning models often possess a large number of parameters and exhibit low operational efficiency. This combination hinders real-time performance and impedes the widespread adoption of these applications (Qiu et al., 2021).

To address these challenges, we propose HA-FuseNet, an end-to-end motor imagery EEG classification network. HA-FuseNet enhances feature extraction efficacy via multi-scale dense connectivity, a hybrid attention mechanism, and a global self-attention module. Concurrently, its lightweight architecture reduces computational overhead, thereby improving real-time performance. Experimental results demonstrate that HA-FuseNet significantly outperforms mainstream benchmark models in both intra-subject and inter-subject classification tasks. This study offers a novel solution that advances the practical application of motor imagery-based brain-computer interface systems.

## 2 Related research

The rapid advancement of deep learning has led to its successful application in motor imagery electroencephalogram (MI-EEG) classification, yielding promising results. Deep neural network architectures, particularly Convolutional Neural Networks (CNNs) and Recurrent Neural Networks (RNNs), are widely employed for Motor Imagery EEG signal classification. Convolutional Neural Networks (CNNs), among the most prevalent deep learning architectures, excel at capturing local signal features and hierarchically extracting higher-level abstract representations as network depth increases. In MI classification tasks, CNNs demonstrate versatility by processing both transformed representations (e.g., time-frequency maps, spatial-frequency maps, spatio-temporal-spectral representations) and raw EEG data. Schirrmeister et al. (2017) introduced the ShallowConvNet and DeepConvNet architectures, which classify EEG signals without manual feature extraction by utilizing axial convolutional layers in place of traditional ones. Lawhern et al. (2018) developed EEGNet, incorporating deep convolution and separable convolution to establish a compact and generalized architecture. Building upon EEGNet, Riyad et al. (2021) proposed MI-EEGNet, specifically optimized for the MI classification task. Mane et al. (2021) introduced FBCNet, integrating the FBCSP algorithm concept. FBCNet filters raw EEG through multiple narrowband filters to derive multi-band representations of the signal. Zhang et al. (2021) employed a multi-branch architecture for multi-scale feature extraction and utilized residual connections to accelerate network training. To capture long-term dependencies in EEG signals, Song et al. (2022) integrated the Transformer model (Vaswani et al., 2017) with a CNN, processing sequences output directly from temporal and spatial convolutional layers. Musallam et al. (2021) developed a ResNet-based model (He et al., 2016) specifically for MI-EEG classification. Miao et al. (2023) proposed a lightweight attention module tailored for EEG signal decoding, enhancing the performance of models like ShallowConvNet and EEGNet. Hsu and Cheng (2023) introduced a wavelet-based time-frequency attention mechanism. This mechanism weights feature maps using both deep attention and time-frequency attention derived from spectrograms. Wang et al. (2023a) segmented EEG signals into two bands (4–16 Hz and 16–40 Hz) and proposed a cross-frequency interaction module. This module enhances features via element-wise addition and average pooling. Notably, this approach exhibits a strong dependence on frequency band filtering. Liu et al. (2023) integrated Squeeze-and-Excitation (SE) modules within three parallel CNN branches, proposing a filtering-free approach.

Recurrent Neural Networks (RNNs) are a class of architectures specifically designed for sequential data processing. Characterized by recurrent structures with feedback connections, they accumulate temporal information and are particularly suited for time-dependent data. Common RNN variants include Long Short-Term Memory (LSTM) and Gated Recurrent Unit (GRU). LSTM, a prominent RNN variant, addresses the vanishing gradient problem inherent in traditional RNNs, enabling effective capture of long-term dependencies. While widely used in natural language processing, LSTM is also effective for processing EEG sequence data in MI tasks and extracting embedded temporal information. Pei et al. (2021) modeled EEG as a multiplexed tensor, fusing multi-band features via tensor decomposition. This approach avoids manual band filtering and enhances cross-subject MI classification performance. Ma et al. (2018) utilized sliding windows for EEG data augmentation prior to LSTM-based MI classification. Wang et al. (2018) developed an LSTM model incorporating 1D aggregation approximation for MI-EEG classification. Effectively modeling long-range dependencies within EEG sequences is crucial for enhancing classification performance. Pei et al. (2025a) proposed that leveraging joint time-frequency dependencies significantly enhances a model’s ability to capture complex EEG patterns. Kumar et al. (2019) introduced a hybrid model integrating Common Spatial Pattern (CSP) feature extraction, Linear Discriminant Analysis (LDA) for dimensionality reduction, and Support Vector Machine (SVM) classification with LSTM. This model achieved accuracies of 68.19 and 82.52% on the GigaDB (Cho et al., 2017) and BCI Competition IV-1 datasets, respectively. Wang et al. (2023b) designed an LSTM-2DCNN hybrid model. In this model, EEG slices are first processed by the LSTM, followed by spatio-temporal feature extraction using 2D convolution.

Despite achieving considerable success in motor imagery (MI) classification tasks by leveraging the powerful feature extraction capabilities of deep neural networks (DNNs), existing methods face several key limitations in practical implementation: (1) DNNs often possess a large number of parameters, resulting in low operational efficiency, increased susceptibility to overfitting, and a reliance on data augmentation techniques that compound computational demands; (2) Due to the inherent non-stationarity and subject-specificity of EEG signals, the performance of many methods exhibits high inter-subject variability, often achieving high accuracy on some individuals but significantly lower and less stable results on others; (3) Some methods depend on high spatial resolution (electrode density) or specific frequency band filtering. However, crucial EEG information is often distributed across a broad frequency spectrum. Consequently, strict band filtering can disrupt intrinsic data relationships and discard valuable information; (4) Furthermore, real-world EEG acquisition devices often lack ideal conditions, such as high spatial resolution.

To address these challenges, we propose the HA-FuseNet model. HA-FuseNet optimizes feature representation through feature fusion and attention mechanisms, while achieving efficient classification via lightweight design.

## 3 Methods

### 3.1 HA-FuseNet: an end-to-end classification network based on feature fusion and attention mechanisms

**Figure 1:**
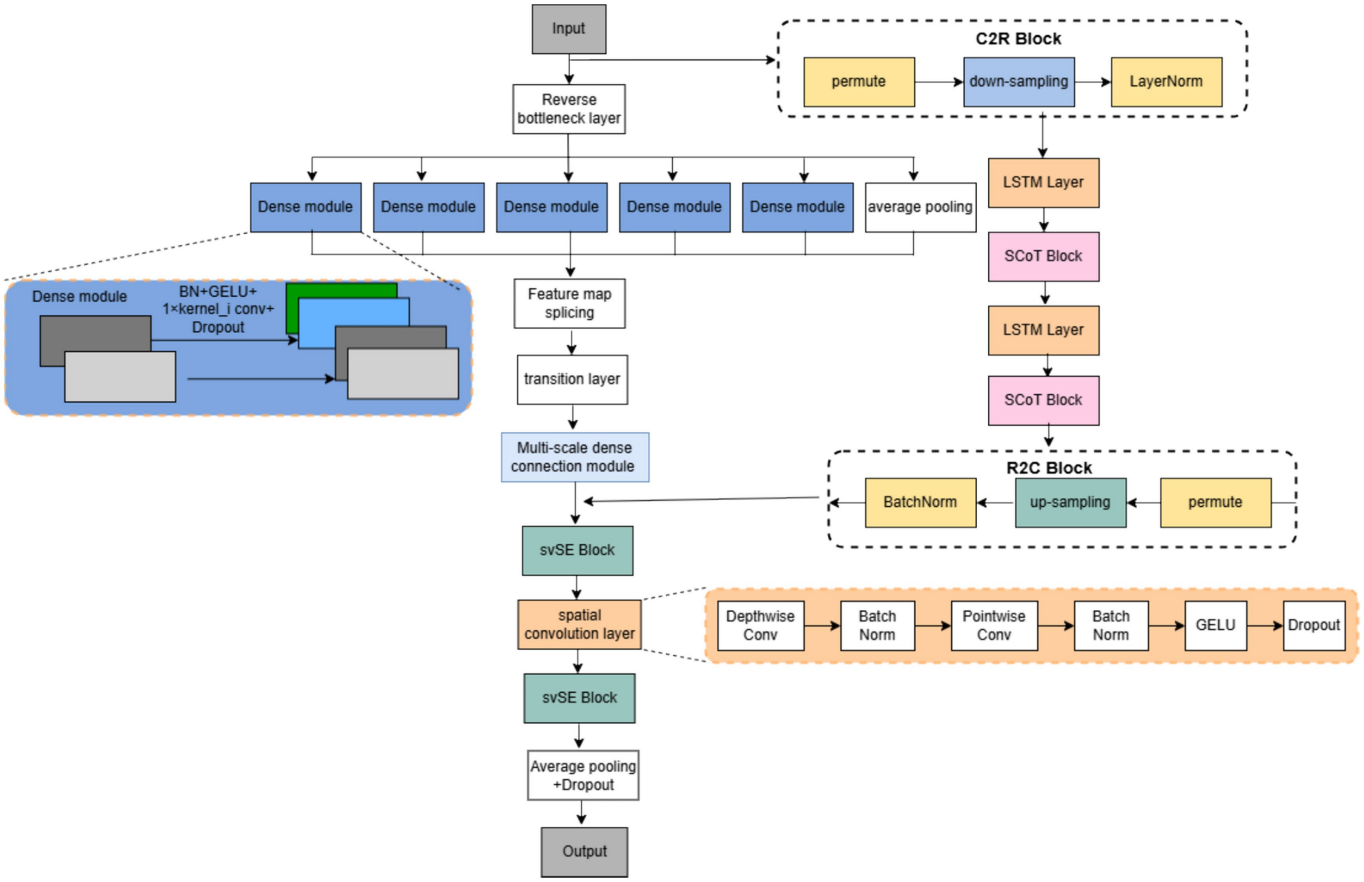
HA-FuseNet architecture.

Multi-domain feature fusion has been established as an effective paradigm for enhancing EEG decoding performance. Specifically, fusing features from the time domain, frequency domain, and nonlinear dynamics domains, and selecting complementary information via attentional weighting, has been demonstrated to outperform single-domain features in EEG-based emotion recognition tasks (Wang et al., 2024). Building upon this paradigm, we construct HA-FuseNet, an end-to-end classification network leveraging feature fusion and attention mechanisms. The architecture of HA-FuseNet is illustrated in Figure 1. HA-FuseNet performs feature extraction, fusion, and final classification prediction utilizing two specialized sub-networks. These sub-networks are: (1) DIS-Net, a convolutional neural network (CNN) based architecture, and (2) LS-Net, based on a Long Short-Term Memory network (LSTM). DIS-Net is designed to extract local spatio-temporal features, while LS-Net captures global spatio-temporal dependencies and long-range contextual information. Therefore, fusing features from DIS-Net and LS-Net leverages their complementary nature: DIS-Net’s local feature representations synergistically combine with LS-Net’s global contextual dependencies. This integration effectively establishes comprehensive spatio-temporal relationships while utilizing fine-grained local information, leading to enhanced classification accuracy.

DIS-Net enhances spatio-temporal feature fusion across depth dimensions via inverted bottleneck layers and incorporates multi-scale dense connectivity by integrating dense connections into the Inception architecture. This design concurrently exploits shallow EEG features and high-level semantics during multi-scale feature extraction, enabling more comprehensive EEG feature representation, and employs an svSE hybrid attention module to selectively emphasize salient features. The svSE module implements spatio-temporal feature decoupling and leverages EEG signal variance for targeted feature enhancement.

LS-Net captures long-term dependencies within the spatio-temporal domain. Specifically, it utilizes LSTM to model long short-term temporal dependencies, and incorporates the SCoT global attention module to acquire global contextual information across the spatio-temporal domain. The two sub-networks interact bidirectionally via the C2R and R2C modules, facilitating feature fusion along the depth dimension. Following fusion, spatial features are extracted using a depthwise separable convolutional layer, reducing computational burden. Finally, classification prediction is performed by a fully connected layer. Collectively, HA-FuseNet enables more comprehensive extraction of discriminative features from MI-EEG signals, leading to improved classification performance.

### 3.2 DIS-Net network

DIS-Net first employs inverted bottleneck layers to expand receptive fields depthwise while facilitating temporal feature fusion. Second, two multi-scale dense connectivity modules extract temporal characteristics, where branch-specific kernel sizes are adapted to the EEG sampling frequency for scale-specific temporal/frequency feature extraction. These modules fuse shallow and deep features depthwise, integrating both low-level details and high-level semantics. Third, a hybrid svSE attention module follows temporal/spatial convolutions to dually recalibrate feature importance, mitigating interference from redundant information and noise to enhance MI-EEG classification accuracy. Fourth, spatial features are extracted via axial depthwise separable convolutional layers. Finally, predictions are generated through pooling, flattening, and Softmax operations.

**Figure 2:**
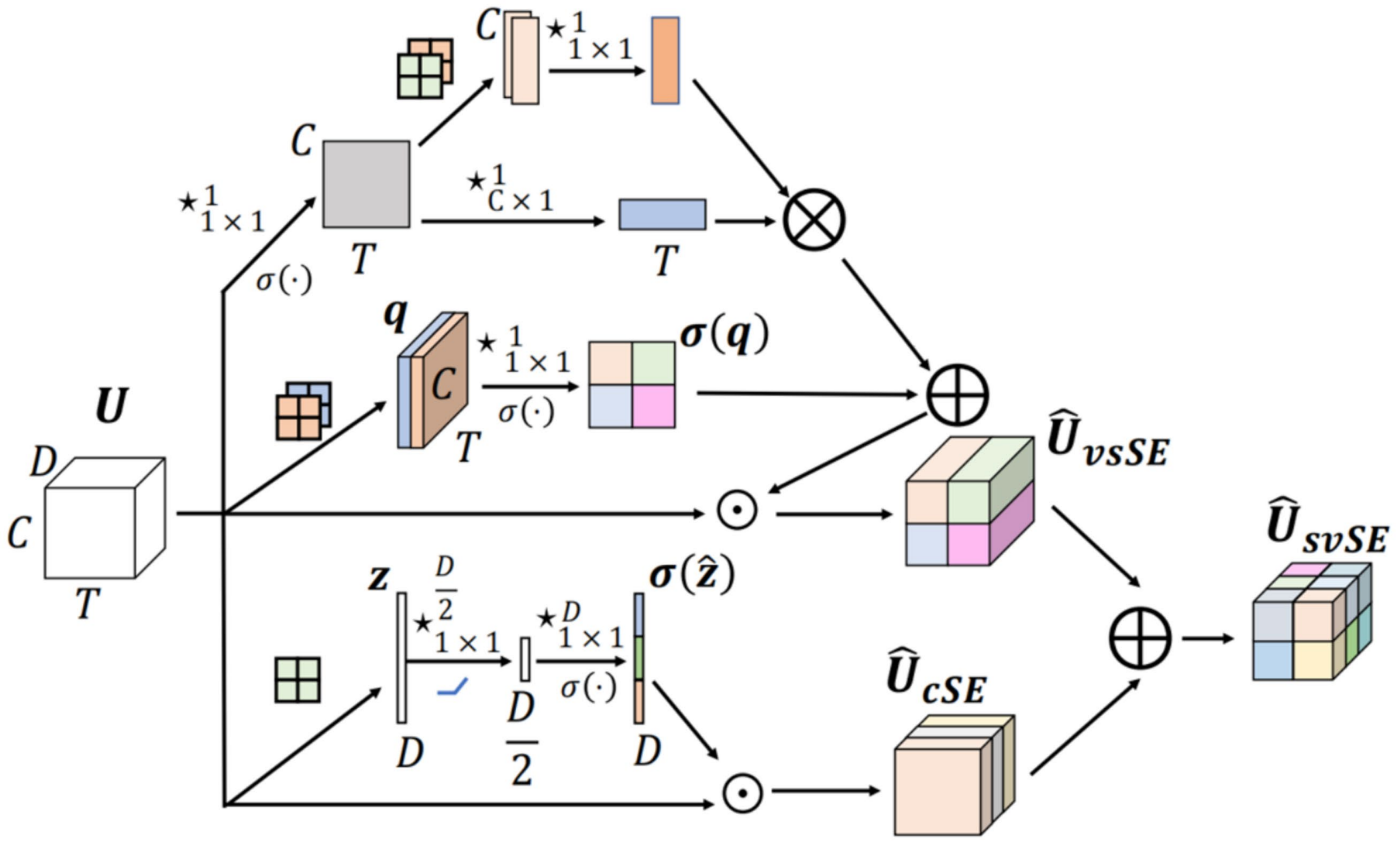
svSE structure.

The svSE (Separate Variance-Informed Spatial and Channel Squeeze-and-Excitation) module is an enhancement of the scSE (Spatial and Channel Squeeze-and-Excitation) module. The scSE module integrates a channel attention mechanism with a spatial attention mechanism, building upon SENet by introducing two sub-modules: the Channel Squeeze-and-Excitation (cSE) module and the Spatial Squeeze-and-Excitation (sSE) module. In the scSE module, these sub-modules process the input features in parallel, apply spatial and channel-wise weighting respectively, and fuse the resulting feature maps. The proposed svSE module is a plug-and-play attention mechanism distinctly designed for MI-EEG classification tasks. It enhances characterization of time-varying EEG features by incorporating variance information, adapts to the low spatio-temporal correlation of EEG signals through axial spatio-temporal attention, and reduces computational overhead while emphasizing discriminative features across diverse data distributions (Pei et al., 2025b). The structure is shown in Figure 2.

For the cSE module, global max pooling is used in place of global average pooling to highlight salient features and generate the channel weight map $A{\mathrm{tt}}_{c}\in R^{D\times 1\times 1}$ . Regarding the sSE module, we propose two improvements; the weights obtained from both methods are combined to produce the final output.

1. For input $X\in R^{D\times C\times T}$ , global mean pooling and global variance computation operations in the depth dimension are used instead of the compression operation in the original module to obtain $X_{\text{pool}}\in R^{2\times C\times T}$ . Subsequently, the feature maps are aggregated in the depth dimension by 1 × 1 convolution, and the resulting weight map $A{\mathrm{tt}}_{v}\in R^{1\times C\times T}$ is obtained to better characterize the temporal variability of the EEG signal;
2. Considering the low correlation of spatio-temporal weights in the EEG signal, features are extracted in two dimensions to obtain the axial attention. For input $X\in R^{D\times C\times T}$ , in the spatial dimension, first a deep compression operation using 1 × 1 convolution is performed to obtain $X_{\mathrm{sf}}\in R^{1\times C\times T}$ .followed by average pooling and maximum pooling on the temporal dimension to obtain two feature maps to obtain $X_{\text{spool}}\in R^{2\times C\times 1}$ . and fusion of these two feature maps by 1 × 1 convolution to obtain $X_{s}\in R^{1\times C\times 1}$ . For the temporal dimension, a convolution operation on the spatial dimension is performed in order to obtain the temporal weight $X_{t}\in R^{1\times 1\times T}$ . Finally, the spatial weights and temporal weights are multiplied by Kronecker product (Kronecker) to recover the dimensionality and obtain the final weight map $A{\mathrm{tt}}_{s}\in R^{1\times C\times T}$ .

where D denotes the input depth, C the channel dimension and T the time dimension. The Softmax activation function is adopted in place of the Sigmoid function to better leverage global context. The complete formulation of the svSE module is given in Equation (1).


(1) $$\begin{array}{l} A{\mathrm{tt}}_{\mathrm{vs}}=A{\mathrm{tt}}_{v}⊕A{\mathrm{tt}}_{s}\\ X_{1}=E\text{xpand}(A{\mathrm{tt}}_{\mathrm{vs}})⊗X\\ X_{2}=E\text{xpand}(A{\mathrm{tt}}_{c})⊗X\\ X_{\text{svSE}}=X_{1}⊕X_{2} \end{array}$$

Where ⊕ denotes element-wise summation, ⊗ denotes element-wise multiplication, and $X_{\mathrm{sv}\mathrm{SE}}$ is the weighted output.

### 3.3 LS-Net network

**Figure 3:**
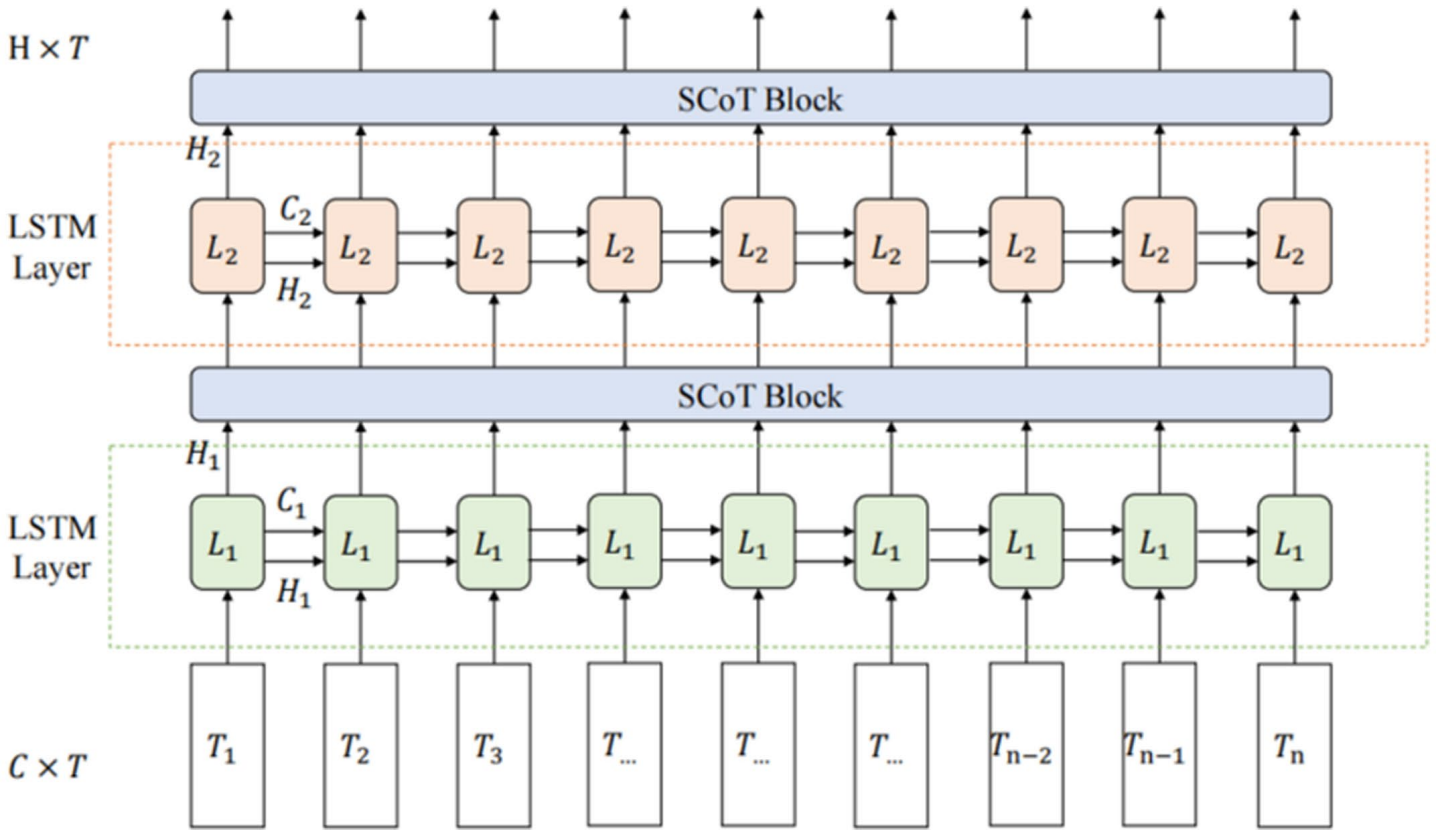
LS-Net structure.

The network architecture combining LSTM with SCoT, shown in Figure 3, is termed LS-Net (LSTM-SCoT Net).

The input of LS-Net is a tensor $X\in R^{C\times T}$ , where C represents the number of channels (features per time sample) and T represents the time sequence length (number of temporal sampling points). During processing, the LSTM sequentially processes each time point in the input sequence, and the output from each LSTM layer is a tensor $Y\in R^{H\times T}$ , where H is the hidden state dimension (size). After processing through each Layer, Y is computed by the SCoT module for global spatio-temporal self-attention. Although H originates from the LSTM’s hidden state dimension, within the SCoT module, it is treated as a channel dimension. This processing integrates the features derived from the LSTM output, enhancing the network’s capacity to capture global spatio-temporal dependencies within the input EEG signal.

While Long Short-Term Memory (LSTM) networks can capture long-term dependencies in sequential data to some extent, EEG signals possess global spatial characteristics as well as long-range temporal dependencies. This characteristic makes LSTM insufficient for fully mining and integrating dependency information across the global spatio-temporal domain. Therefore, building upon improvements derived from two global self-attention mechanisms Non-local (Wang et al., 2018) and Contextual Transformer (CoT) (Li et al., 2022), this work proposes the SCoT module, specifically designed to address the characteristics of EEG signals. It aims to more comprehensively capture long-term dependencies within the spatio-temporal domain, thereby enhancing the model’s global modeling capability.

**Figure 4:**
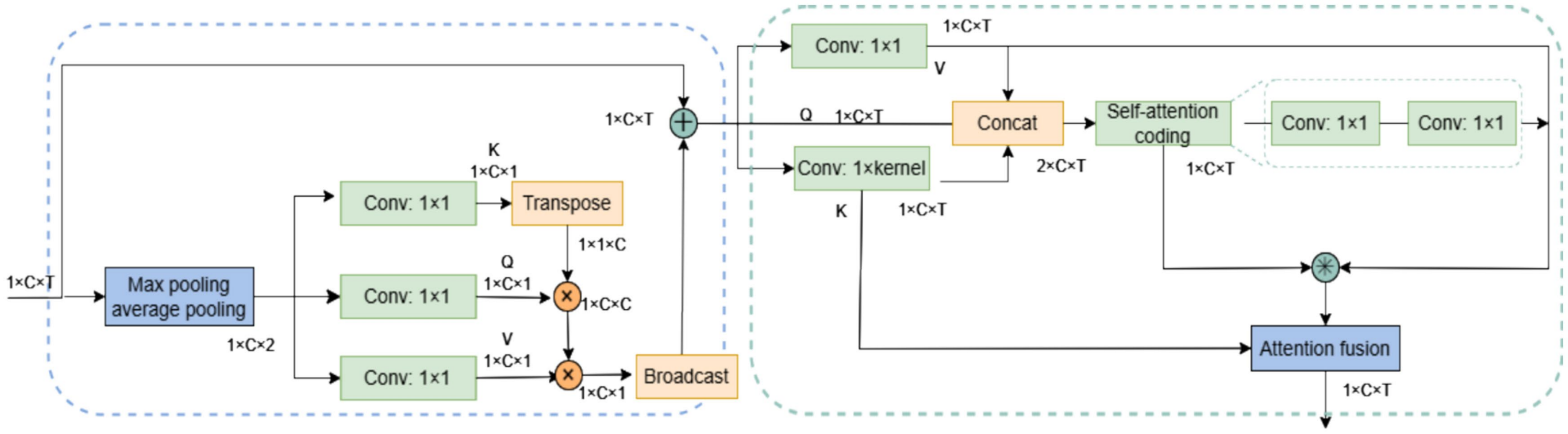
SCoT structure.

The SCoT attention module adopts a step-by-step strategy to compute global self-attention across the spatio-temporal domain. For spatial information, characterized by weak local correlation and limited data size, the Non-local module is adapted for spatial self-attention computation. Conversely, for temporal information exhibiting strong local correlation and large data size, computation of temporal self-attention is enhanced using Contextual Transformer (CoT). The overall structure of the SCoT module is illustrated in Figure 4.

SCoT computes the global spatio-temporal attention of the EEG signal in two processes. First, the spatial self-attention in the spatial domain is computed, and after the inputs are weighted using the spatial self-attention, the spatio-temporal domain self-attention is computed based on the weighted data. The spatial self-attention can allocate more attention to the important channels and highlight the relative importance of different channels in the case of weak local correlation. The spatio-temporal domain self-attention leverages the local correlation within the time series data and the spatial feature information that has been enhanced by the spatial self-attention weighting, so as to capture the global spatio-temporal dependence in the EEG signal more comprehensively and precisely, while also calibrating the attention obtained in the previous step. Additionally, this two-stage computation helps reduce computational complexity. The overall process of calculating the attention in the SCoT module is shown in Equation (2).


(2) $$\mathrm{SC}oT(X)=\mathrm{At}t_{\mathrm{st}}({\mathrm{Att}}_{s}(X)),X\in R^{1\times C\times T}$$

where X is the EEG signal input, C denotes the number of channels, T signifies the number of time steps, *Att*_s_ is the spatial self-attention module, and *Att*_st_ is the spatio-temporal self-attention module.

**Figure 5:**
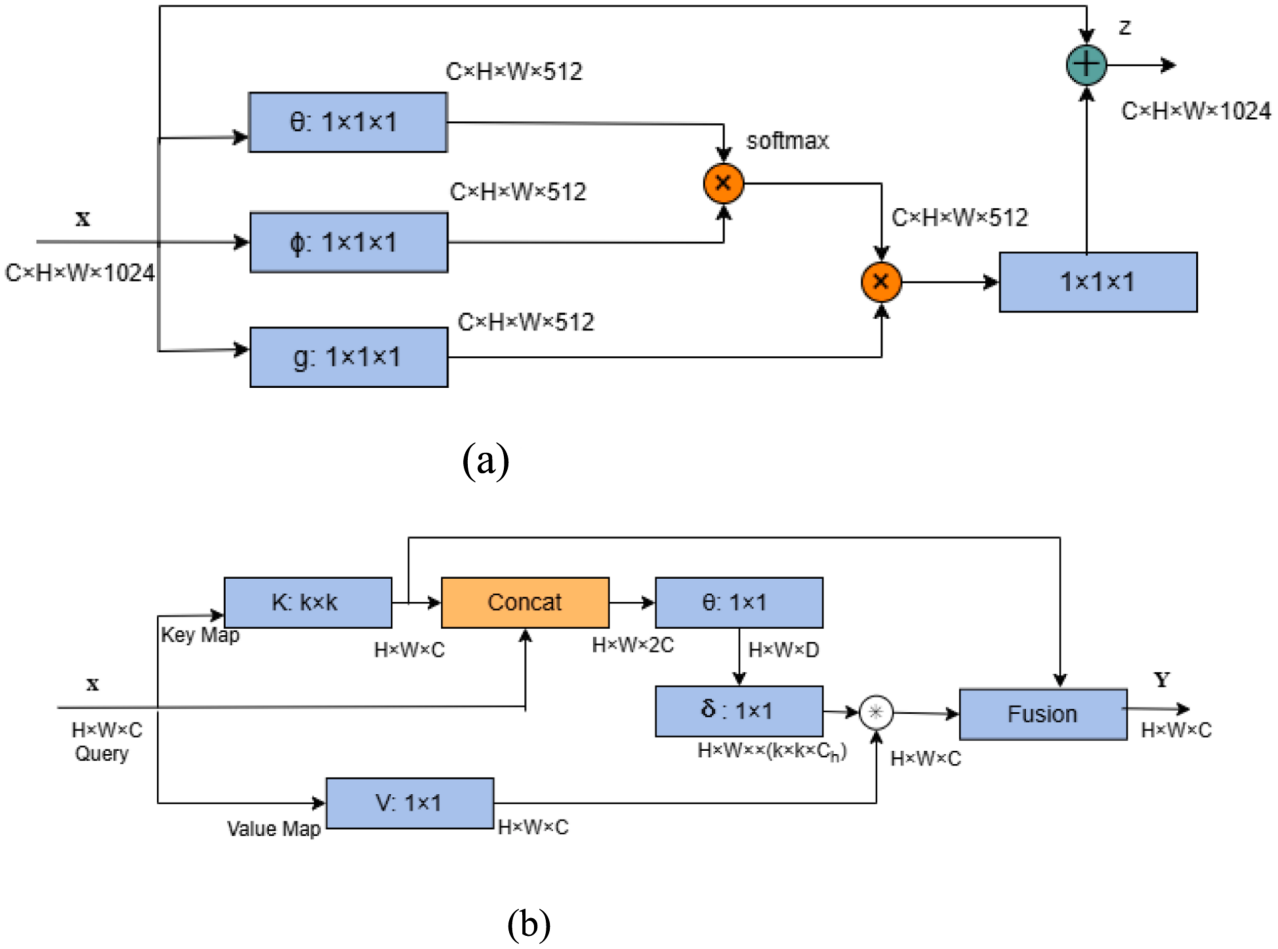
Two types of global self-attention. **(a)** Non-local structure. **(b)** CoT structure.

Non-local is a classical model that applies self-attention to computer vision, capturing dependencies between any two positions in feature maps. Its structure is shown in Figure 5a. For input $X\in R^{C\times H\times W}$ , the number of channels is first compressed from C to $\frac{C}{2}$ by three 1×1 convolutions, resulting in three feature maps $X_{\theta }⊥X_{\phi }$ and $X_{g}$ representing the query, key, and value matrices, respectively. Subsequently, $X_{\theta }⊥X_{\phi }⊥X_{g}$ are flattened, and the similarity matrices of $X_{\theta }$ and $X_{\phi }$ are computed by the dot-product operation S . The matrices represent the associations between the positions in the input feature maps. Next, the Softmax function is used to normalize the similarity matrix. S so that it is transformed into a probability distribution and multiplied with the value matrix $X_{g}$ to obtain the result matrix $Y\in R^{\frac{C}{2}\times H\times W}$ . Finally, the dimension of Y is recovered to be the same as that of X by 1×1 convolution to obtain the weighted result by element-by-element summation. Non-local captures global dependencies through direct computation of long-range interactions, but incurs high computational costs with large-scale data.

CoT considers the problem that Non-local ignores the neighboring key context information and proposes a way to integrate the context information mining capability into the self-attention mechanism, whose structure is shown in Figure 5b. For input $X\in R^{C\times H\times W}$ , CoT primarily involves obtaining the static context information of the feature map using 3×3 convolution as the key matrix K . Meanwhile, the query matrix Q is directly obtained by 1×1 convolution to the value matrix V , and then K and Q are aggregated in depth dimension and the attention weight map A is obtained by two consecutive 1×1 convolution, and the feature map is obtained by the multiplication of the attention weight map A and the value matrix V to the dynamic context information. Finally, the static and dynamic context features are fused.

### 3.4 Improved lightweight network based on GhostNet

**Figure 6:**
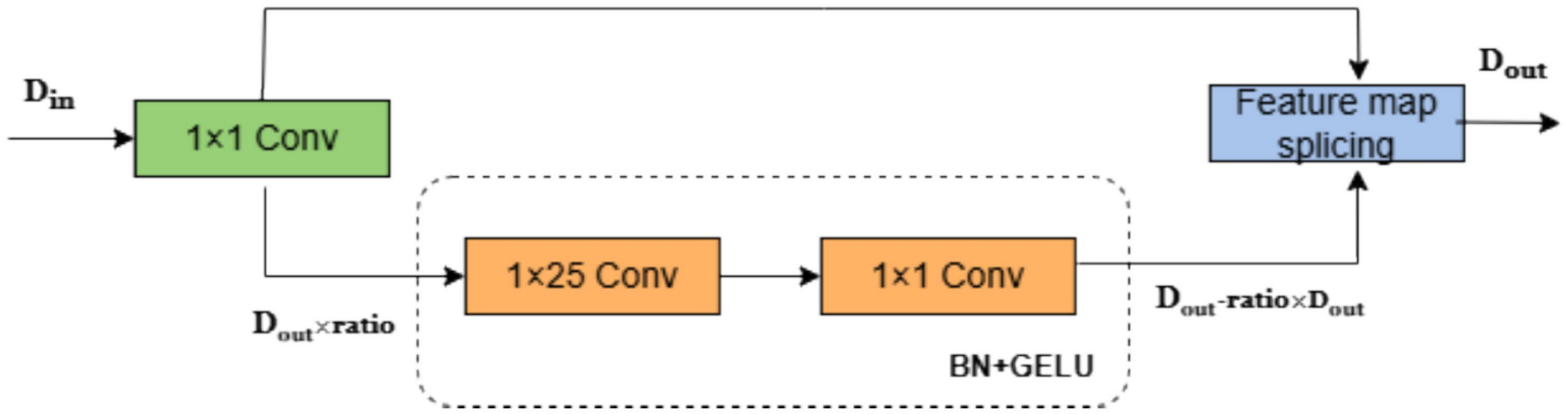
SG network structure diagram.

To optimize real-time performance of HA-FuseNet, this paper enhances the Ghost module and constructs the SG module (Separable Ghost Module) using depthwise separable convolution. As shown in Figure 6, the SG module strengthens feature map interactions through two convolutional layers (depthwise and pointwise convolutions). The dotted box indicates the replacement for the original convolutional layer, where *D*_in_ denotes input channels, *D*_out_ denotes output channels, and ratio is the compression hyperparameter.

In the SG module, the number of input feature maps is *D*_out_ × ratio, and the number of output feature maps is *D*_out_ - ratio × *D*_out_ to maintain consistent output channel dimensions. The convolution kernel size is determined based on sampling frequency characteristics. For clarity, taking the temporal feature extraction convolution as an example, its kernel size is set to 1 × 25. For the BCI Competition IV Dataset 2A sampled at 250 Hz, this corresponds to a 100-millisecond temporal window for feature extraction. In the lightweight convolutional layer: (1) Features are first processed by a 1 × 25 depthwise separable convolution without altering channel count; (2) After batch normalization, features interact via a 1 × 1 pointwise convolution that modifies channel dimensions; (3) Finally processed through batch normalization with GELU activation.

The number of input feature maps for the lightweight transformed convolutional layer is *D*_out_ × ratio and the number of output feature maps is *D*_out_ – ratio × *D*_out_ to ensure that the number of output feature maps is unbiased. The number of output feature maps passed directly from the standard convolution is *D*_out_ × ratio, and all feature maps are aggregated in the depth dimension to obtain the final output.

## 4 Model training and evaluation

### 4.1 Experimental equipment

The proposed HA-FuseNet network was trained on a laboratory server, and the software/hardware environment used is shown in Table 1.

**Table 1:** Experimental environment.

Software/hardware name	Model/version
OS	Ubuntu 20.04.6 LTS
GPU	NVIDIA GeForce RTX 3090
CPU	Intel(R) Xeon(R) Gold 5218R CPU @ 2.10GHz
RAM	128G
GPU Memory	24G
CUDA	11.8
Python	3.11.5
Pytorch	2.0.1
MNE	1.6.0
Numpy	1.26.3

### 4.2 Dataset description

Several public EEG datasets exist for motor imagery research. We primarily utilized BCI Competition IV Dataset 2A (Brunner et al., 2008) for model training and evaluation. This dataset contains motor imagery EEG (MI-EEG) recordings from nine subjects, comprising four motor imagery tasks: left hand, right hand, feet, and tongue movements.

Each subject completed two sessions recorded on different dates, designated, respectively, as training set (T) and test set (E). Data were stored in GDF format, with each subject having two files (e.g., Subject 1 has A01T.gdf and A01E.gdf). The training set file (A01T.gdf) includes annotation labels, while the test set file (A01E.gdf) requires separate label information provided in the corresponding A01E.mat file.

### 4.3 Data pre-processing

EEG signals are characterized by low signal-to-noise ratio, non-stationarity, and spatial variability, with typically limited sample sizes. Although preprocessing often benefits classification tasks, this study develops an end-to-end network requiring minimal preprocessing. The applied preprocessing includes.

(1) Data extraction and slicing

The raw MI-EEG signals are stored in .gdf format files. Besides target EEG signals, these files contain electrooculography (EOG) signals, data gaps (represented as missing values), and non-task-related events. Consequently, specialized extraction procedures are required.

**Figure 7:**
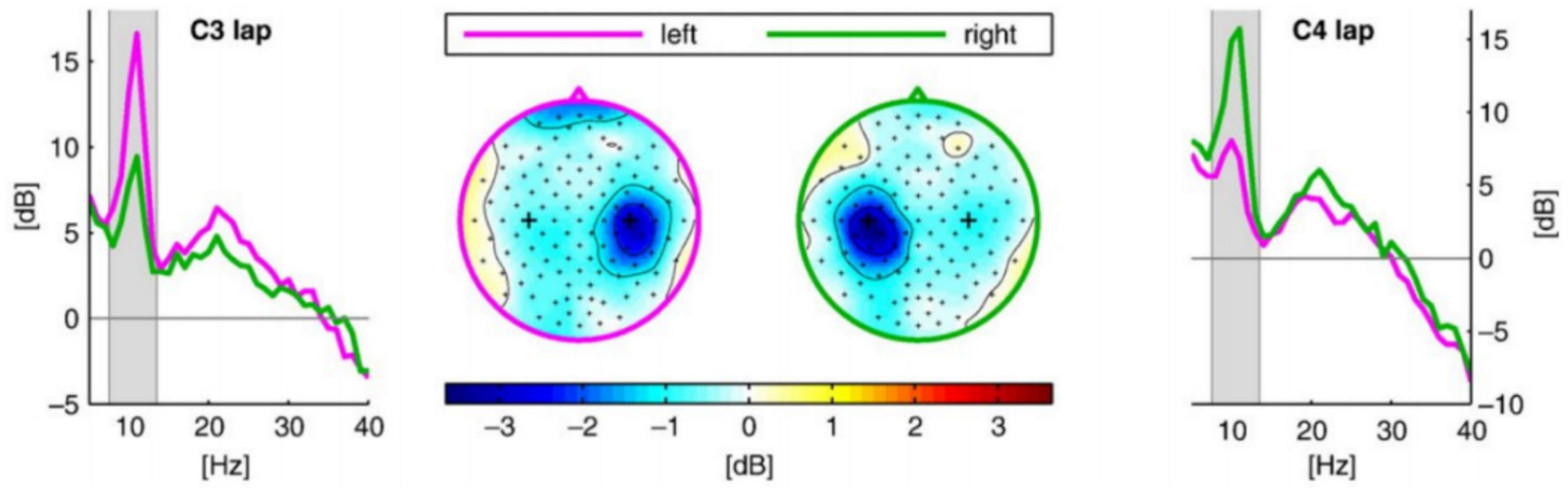
ERD phenomena in left-and right-handed motor imagery.

First, electrooculography (EOG) channels were excluded, retaining only electroencephalography (EEG) channels. For missing values (encoded as NaN) marking run intervals, channel-wise mean imputation was applied to ensure data continuity and integrity;

Second, events directly related to the motor imagery task were screened and extracted, and the relevant time periods were sliced. For the BCI Competition IV Dataset 2A dataset, the directly related events were both 4-class and 2-class motor imagery task events, The start of the task was marked by a cue signal (Cue). We extracted the data segment from the 1st to the 4th second after the appearance of the Cue (corresponding to the 3rd to 6th second of the trial cycle) as the EEG signal corresponding to the motor imagery task duration for subsequent analysis. Motor imagery-related EEG activity (e.g., ERDs/ERSs of Mu/Beta rhythms) usually appears significantly only after a short delay after task onset. The choice of t = 3 s onset circumvents the visual evoked artifacts at the moment of Cue appearance and ensures that the extracted signals more purely reflect the motor imagery itself. Figure 7 shows a plot of ERD phenomena for right-and left-handed motor imagery, showing that ERD phenomena at the C3/C4 electrodes gradually increased after imagery onset (Blankertz et al., 2010).

(2) Normalization

The purpose of normalization is to standardize feature scaling, eliminating adverse effects caused by anomalous samples while improving model training efficiency and stability. Z-score standardization and min-max normalization are classic algorithms in EEG signal preprocessing.

Z-score standardized data follows a standard normal distribution. The operation is formalized in Equation (3).


(3) $$x=\frac{x-\mu }{\sigma }$$

Where *x* is the original data, μ denotes the mean value of *x*, and σ denotes the standard deviation of x.

The operation process of the maximum-minimum normalization is shown in Equation (4), which is a linear transformation operation that maps the data to the interval [0, 1]. where X represents a set of channel data.


(4) $$x=\frac{x-\min(X)}{\max(X)-\min(X)}$$

Comparative analysis shows that Z-score, based on mean and standard deviation, is insensitive to outliers. Although transient noise or artifacts (e.g., electromyographic interference) may be present in the EEG signal, the linear transformation of Z-score preserves the overall distributional properties of the data. Maximum-minimum normalization relies on maximum and minimum values. However, if extreme values exist in the signal (e.g., transient high amplitude due to poor electrode contact), the normalized data will be compressed to a very small range, resulting in distorted information. Therefore, to preserve the characteristics of the EEG signal as much as possible, we employed the Z-Score method for EEG signal normalization to improve the speed and stability of model training.

### 4.4 Evaluation metrics

(1) Evaluation metrics

The study mainly uses Accuracy (Acc), Kappa Consistency Coefficient (Kappa), and Standard Deviation (SD) as the evaluation metrics of the model. Among them, Accuracy, a commonly used evaluation metric in classification tasks, is used to measure the proportion of model predictions that match the true labels, which is calculated as the proportion of samples that are correctly classified out of the total samples.

Kappa consistency coefficient is used to measure the degree of consistency between the model prediction results and the real labels, which is especially effective in the case of class imbalance or random guessing with some effect. The Kappa coefficient is computed based on the confusion matrix, and its computational formula is shown in Equation (5).


(5) $$K\text{appa}=\frac{P_{0}-P_{e}}{1-P_{e}}$$

Where *P*_0_ is the Observed Proportion of Agreement, i.e., the accuracy rate, and *P*_e_ is the Expected Proportion of Agreement by Chance. Assuming the total number of samples is N, the total number of categories is c, the number of real samples for the iᵗʰ action class is *x*_i_, and the number of predicted samples is *P*_i_, the formula for *P*_e_ is shown in Equation (6).


(6) $$P_{e}=\frac{\sum_{i=1}^{c}x_{i}\times P_{i}}{N^{2}}$$

The Kappa coefficient ranges from −1 to 1, usually greater than 0. The larger the Kappa value, the higher the degree of consistency, and when the Kappa value is located in the interval of [0.61, 0.80], it means that the prediction has a high degree of consistency with the real one.

The standard deviation is the standard deviation of the accuracy rate, which is used to measure the stability ability of the model between different subjects, and the smaller its value, indicating that the accuracy rate of different subjects is more similar, and the performance of the model between different subjects is more stable. The standard deviation of the accuracy rate is calculated as shown in Equation (7).


(7) $$\mathrm{SD}=\sqrt{\frac{1}{N-1}\sum_{i=1}^{N}{\left(x_{i}-\bar{x}\right)}^{2}}$$

Where N denotes the number of subjects, *x*_i_ denotes the accuracy rate of the iᵗʰ subject, and $\bar{x}$ is the average of the accuracy rates of N subjects.

(2) Loss function

The categorical cross-entropy loss function is employed, which quantifies the discrepancy between the predicted probability distribution and the true label distribution. Minimizing this loss encourages the predicted distribution to converge toward the target distribution. The cross-entropy loss function is defined as Equation (8):


(8) $$L=-\sum_{i=1}^{C}y_{i}\log({\hat{y}}_{i})$$

Where C represents the number of classes, *y*_i_ denotes the true class label, and ${\hat{y}}_{i}$ represents the predicted probability for class i. This loss function penalizes the model’s predictions based on the true labels, thereby driving the model to learn more accurate predictions.

(3) Training parameters

The Adam optimization algorithm was employed. For the BCI Competition IV Dataset 2A, the batch size was set to 32. The experiments were run for 300 epochs. The initial learning rate was set to 1 × 10^−3^ and the weight decay coefficient was set to 5 × 10^−3^.

## 5 Results and analysis

This paper presents a comprehensive experimental evaluation of the proposed HA-FuseNet model on the BCI Competition IV Dataset 2A dataset, including within-subject (Subject Dependent) and between-subject (Subject Independent) comparison experiments. The effectiveness and cross-subject generalization ability of the HA-FuseNet model in motor imagery electroencephalography (MI-EEG) classification tasks were verified by comparing its performance with that of mainstream models. In addition, a lightweight version of HA-FuseNet [HA-FuseNet (SG)] is experimentally evaluated in this paper to explore its trade-off between efficiency and performance. All experimental results of the baseline model in this paper are based on the replication of the official open source code on the BCI Competition IV Dataset 2A dataset.

### 5.1 Ablation experiments

To explore the effectiveness of each proposed module and study the influence of different modules on HA-FuseNet’s classification performance, the thesis conducts ablation experiments. Starting from the baseline model and following the order of model construction, the study experimentally verifies the impact of incrementally adding new modules. The paper defines models with varying structures as follows:

(1) Inception: The base Inception model with three Inception modules modified with convolutional kernels for the characteristics of EEG signals.
(2) Base-Inception: Inception + Bottleneck, i.e., BaseNet proposed in the thesis. This introduces a reversed bottleneck layer on top of the Inception model and adjusts the number of branches, activation functions, etc. The convolutional kernel size is also adapted for EEG signal characteristics.
(3) BI+Dense: Inception + Bottleneck + Dense Block, i.e., DI-Net proposed in the paper. This introduces a dense connection module on the basis of BaseNet.
(4) DI + svSE: Inception + Bottleneck + Dense Block + svSE, i.e., DIS-Net proposed in the paper. This introduces an svSE hybrid attention module on the basis of DI-Net.
(5) DIS + LSTM: Inception + Bottleneck + Dense Block + svSE + LSTM. This introduces an LSTM network on the basis of DIS-Net.
(6) DIS + LSTM+SCoT: Inception + Bottleneck + Dense Block + svSE + LSTM + SCoT, i.e., HA-FuseNet (combining DIS-Net and LS-Net) as proposed in the paper. This introduces an SCoT global self-attention module on top of DIS + LSTM.

Ablation experiments were conducted on the BCI IV 2a dataset, with the results presented in Table 2. The reported accuracy and Kappa coefficient represent the average performance across nine participants. The experimental data show that the architecture combining Inception, Bottleneck, Dense Block, svSE, LSTM, and SCoT–which constitutes the final model proposed in this paper, HA-FuseNet–achieved the best performance on the MI-EEG classification task. It attained an accuracy of 77.89% and a Kappa coefficient of 0.70, with a standard deviation of 10.22. These results demonstrate that HA-FuseNet achieved the best classification performance across different participants, exhibiting relatively low variability in accuracy between participants and indicating robust generalization capabilities.

**Table 2:** Comparison of HA-FuseNet ablation experiment results by module.

Model	ACC (%)	Kappa	SD
Inception	67.40	0.56	16.15
Base-Inception	72.35	0.63	12.27
BI+Dense	74.42	0.65	11.92
DI + svSE	76.16	0.68	11.33
DIS + LSTM	76.78	0.69	10.72
DIS + LSTM+SCoT*	**77.89**	**0.70**	**10.22**

The models proposed in the paper are marked with *. The optimal values in the table are marked with bolding.

As the model gradually increased in complexity, both accuracy and the Kappa coefficient showed an upward trend, while the standard deviation exhibited a downward trend. This demonstrates the effectiveness of each module. Furthermore, BaseNet, obtained by making task-specific refinements to Inception for the MI-EEG classification task, yielded the most significant improvement. This was followed, in order, by the multi-scale dense connection module, the svSE module, the SCoT module, and the LSTM module. In terms of contribution level to the final model, these results suggest that convolutional neural networks (CNNs) may hold a greater advantage over recurrent neural networks (RNNs) for MI-EEG classification tasks.

The experiments have demonstrated the effectiveness of the individual modules proposed in the paper. These modules collectively form the final model, HA-FuseNet, achieving an average accuracy of 77.89% and an average Kappa coefficient of 0.70 on the BCI IV 2a dataset. The full model was used in subsequent experiments.

### 5.2 Intra-subject comparison experiments

The BCI IV 2A dataset has a total of nine subjects, each with an independent training and test set, and the intra-subject comparison experiment means that a model was trained for each subject using the corresponding training and test sets for each subject. Table 3 shows the accuracy rates of different models for within-subject experiments, with the last column being the mean of the accuracy rates for the nine subjects, and Table 4 demonstrates the Kappa consistency coefficients and standard deviation (SD) for each model, where the Kappa consistency coefficients are the mean of the nine subjects, and the standard deviation is the standard deviation of the accuracy rates. To present the data in a more intuitive way, the optimal values in the table are marked with bolding, and the models proposed in the paper are marked with*.

**Table 3:** Comparison of intra-subject experimental results between HA-FuseNet and the benchmark model on the 2A dataset (Acc %).

Model	1	2	3	4	5	6	7	8	9	Average value
Shallow ConvNet	84.03	61.46	**94.10**	**70.83**	73.26	55.90	85.76	**89.24**	**85.42**	77.78
Deep ConvNet	83.68	**65.28**	90.63	69.44	**76.04**	64.58	89.93	79.51	73.61	76.97
EEG Net	78.13	63.54	82.30	60.42	71.88	59.03	72.92	68.06	66.67	69.21
EEG Inception	71.18	48.26	82.29	55.90	64.58	52.43	75.00	85.41	73.61	67.63
EEG Conformer	67.71	55.21	84.72	53.82	75.69	53.47	69.10	71.53	58.68	65.55
LMDA-Net	86.46	60.46	90.97	59.02	69.10	55.90	90.28	81.94	76.04	74.50
HA-FuseNet*	**87.67**	62.85	92.36	67.54	75.00	**65.97**	89.58	81.60	78.47	**77.89**
HA-FuseNet(SG)*	86.81	63.89	92.01	65.54	71.88	62.85	**91.67**	83.68	74.95	77.23

The models proposed in the paper are marked with *. The optimal values in the table are marked with bolding.

**Table 4:** Comparison of intra-subject experimental results between HA-FuseNet and the benchmark model on the 2A dataset (Kappa/SD).

Model	Kappa	SD
Shallow ConvNet (Schirrmeister et al., 2017)	0.69	12.35
Deep ConvNet (Schirrmeister et al., 2017)	0.69	9.22
EEG Net (Lawhern et al., 2018)	0.60	**7.40**
EEG Inception (Zhang et al., 2021)	0.56	12.41
EEG Conformer (Song et al., 2022)	0.53	10.33
LMDA-Net (Miao et al., 2023)	0.65	13.01
HA-FuseNet*	**0.70**	10.22
HA-FuseNet(SG)*	0.69	11.14

The models proposed in the paper are marked with *. The optimal values in the table are marked with bolding.

Experimental result analysis:

(1) Among the benchmark models, ShallowConvNet achieves the highest average accuracy and Kappa value. However, its accuracy standard deviation reaches 12.35, indicating significant performance variation across subjects and relative imbalance;
(2) EEGNet achieves the optimal standard deviation of the accuracy rate, indicating that the difference in the accuracy rate between different subjects is small and the performance is relatively balanced, but the average accuracy rate and Kappa value are 69.21% and 0.60, respectively, which are lower than those of ShallowConvNet, DeepConvNet and LMDA-Net;
(3) LMDA-Net’s average accuracy and Kappa value are only lower than those of ShallowConvNet and DeepConvNet, but it exhibits the highest standard deviation of accuracy, indicating the most pronounced performance difference across subjects. As LMDA-Net builds upon improvements to EEGNet and ShallowConvNet, this variability may primarily stem from its proposed local channel attention and deep attention mechanisms, which exhibit uneven effectiveness in capturing features across different subjects;

Comparing the experimental results of the benchmark models with the proposed model, HA-FuseNet achieves the highest average accuracy and Kappa value and the highest accuracy on subjects 1 and 6, and also achieves good performance for subjects 2 and 4, where the average performance of the other models is poor. Meanwhile, the standard deviation of the accuracy of HA-FuseNet is lower than that of ShallowConvNet with sub-optimal average accuracy and kappa values; HA-FuseNet(SG), utilizing the SG lightweight convolutional module, achieves accuracy and Kappa values second only to those of ShallowConvNet, and the standard deviation of its accuracy is also lower than that of Compared with HA-FuseNet, the average accuracy of HA-FuseNet(SG) only decreases by about 0.66 percentage points.

The experiments demonstrate the effectiveness of HA-FuseNet and HA-FuseNet(SG) in achieving good performance when using small-scale datasets. In addition, the analysis of the experimental results for each model corroborates the theory of previous studies about the excellent performance of shallow networks in MI-EEG classification tasks with small-scale datasets, while the multi-scale dense connectivity enables HA-FuseNet to utilize both the low-level features and the high-level semantic information at multiple scales simultaneously, and at the same time, through global self-attention mechanism and local hybrid attention mechanism for multidimensional weighting, and the extraction of long and short-term dependencies in the time dimension by using LSTM, the combination achieves better performance than the benchmark model. Although F1 values are not shown for each category, the accuracy improvement for Subject 6 in the intra-subject experiment in Table 3 is significant (HA-FuseNet: 65.97% vs. ShallowConvNet: 55.90%), suggesting that the model has increased its ability to recognize low divisibility categories (e.g., foot movements).

### 5.3 Inter-subject comparison experiments

In the inter-subject comparison experiment, for subject i, the training set of all other subjects were combined to form the training set for subject i, as defined in Equation (9), where *Train*_j_ denotes the training set of subject j. The test set of subject i is still used as the test set.


(9) $$Tr{\mathrm{ain}}_{i}=\sum_{j}^{9}{\text{Train}}_{j},i\in (1,2,\ldots ,9),j\in (1,2,\ldots ,9),j\neq i$$

Table 5 demonstrates the comparison of the accuracy results of the inter-subject experiments performed by each model. For example, [Shallow ConvNet, 1] in the table indicates the accuracy achieved by ShallowConvNet when trained on the combined training sets of the other eight subjects and tested on the test set of subject 1. Table 6 demonstrates the comparison of the Kappa coefficient and standard deviation results for the inter-subject experiments. Comparing the experimental data of the benchmark model, the following results can be found:

**Table 5:** Comparison of inter-subject experimental results between HA-FuseNet and the baseline model on the 2A dataset (Acc %).

Model	1	2	3	4	5	6	7	8	9	Average value
Shallow ConvNet	**76.39**	47.92	**88.54**	55.56	57.64	55.21	74.65	**81.25**	72.92	67.79
Deep ConvNet	71.53	50.69	84.72	**61.46**	**69.10**	59.03	75.35	74.31	64.93	67.90
EEG Net	68.75	**56.60**	68.75	61.11	68.75	58.68	73.61	56.60	56.25	63.23
EEG Inception	74.31	51.04	81.60	52.43	56.25	**60.42**	71.18	73.96	**74.31**	66.17
EEG Conformer	52.78	26.04	26.04	50.00	64.24	25.00	26.74	29.51	27.78	36.46
LMDA-Net	72.22	47.22	83.68	55.90	51.74	48.26	71.88	76.04	66.67	63.73
HA-FuseNet*	76.13	52.79	86.89	57.13	60.42	58.49	**76.74**	78.13	70.06	**68.53**

The models proposed in the paper are marked with *. The optimal values in the table are marked with bolding.

**Table 6:** Comparison of inter-subject experimental results between HA-FuseNet and the benchmark model on the 2A dataset (Kappa/SD).

Model	Kappa	SD
Shallow ConvNet (Schirrmeister et al., 2017)	0.57	13.19
Deep ConvNet (Schirrmeister et al., 2017)	0.57	9.54
EEG Net (Lawhern et al., 2018)	0.51	**6.33**
EEG Inception (Zhang et al., 2021)	0.55	10.57
EEG Conformer (Song et al., 2022)	0.15	14.09
LMDA Net (Miao et al., 2023)	0.52	12.53
HA FuseNet*	**0.57**	11.06

The models proposed in the paper are marked with *. The optimal values in the table are marked with bolding.

Experimental result analysis:

(1) Among the benchmark models, DeepConvNet achieves the highest average accuracy and Kappa coefficient, with an accuracy standard deviation of 9.54. It outperformed ShallowConvNet in the inter-subject experiments, potentially because DeepConvNet learns more abstract and high-level features, enabling better adaptation to data differences between subjects;
(2) EEGNet achieved the optimal standard deviation of accuracy, but the average accuracy and Kappa value were 63.23% and 0.51, which were only better than EEGConformer, which indicated that EEGNet had better stability, but the accuracy and consistency of classification were poor;
(3) The average performance of all benchmark models was poor in the cross-subject experiments of subjects 2, 4, and 6. Comparing the experimental data of the benchmark models and the model proposed in the paper, it can be found that the HA-FuseNet proposed in the paper achieves the optimal average accuracy and Kappa coefficient, and the average accuracy is improved by about 0.63% compared with DeepConvNet, and the standard deviation of the accuracy of HA-FuseNet reaches 11.06, which is only higher than that of DeepConvNet by about In addition, the proposed HA-FuseNet outperforms DeepConvNet on subjects 1, 2, 3, 7, 8, and 9, and underperforms DeepConvNet only on subjects 4, 5, and 6; however, it should be noted that HA-FuseNet achieves a more balanced classification accuracy on subjects 2, 4, and 6, which have a poorer average performance of the benchmark model. However, it should be noted that HA-FuseNet achieves a more balanced classification accuracy on subjects 2, 4, and 6, where the average performance of the benchmark model is worse than that of DeepConvNet, and HA-FuseNet achieves a more balanced classification accuracy, which is lower than that of EEGNet on subject 2, DeepConvNet, and EEGNet on subject 4. The experiments confirm that the proposed HA-FuseNet meets the research objectives, as it has a good classification performance and cross-subject generalization ability on the 22-channel BCI IV 2A dataset. The experiments confirm that the proposed HA-FuseNet has good classification performance and cross-subject generalization ability on the BCI IV 2A dataset with 22 channels, which can satisfy the research objectives of the paper.

## 6 Conclusion

This paper proposes an end-to-end classification model, HA-FuseNet, based on feature fusion and attention mechanisms to address the problems of low signal-to-noise ratio, subject-specificity, and overfitting of small-sample data in the classification task of motor imagery electroencephalography (MI-EEG), which effectively fuses shallow detailed features with deeper semantic information. This is achieved through convolutional kernels adapted to the sampling frequency and the incorporation of a dense connectivity mechanism, enhancing the model’s ability to characterize the complex time-frequency characteristics of EEG signals. Information to improve the model’s ability to characterize the complex time-frequency characteristics of EEG signals. The proposed svSE module introduces variance pooling and axial spatio-temporal attention separation to enhance the dynamic perception of important features within non-stationary EEG signals. By combining this with the global context modeling capability of the SCoT module, spatio-temporal dependencies are further optimized. By utilizing axial convolution alongside the improved SG lightweight convolution module, classification accuracy is maintained while reducing the number of parameters. Experiments on the BCI Competition IV Dataset 2A dataset show that HA-FuseNet achieves an average accuracy of 77.89% in intra-subject experiments, which is an improvement of about 8.42% compared with mainstream models such as EEGNet; the average accuracy in inter-subject experiments is 68.53%, significantly outperforming the comparison methods.

Despite the significant progress of HA-FuseNet in the MI-EEG classification task, there is still room for improvement. In the future, multi-modal data such as near-infrared spectroscopy (NIRS) or electromyography (EMG) can be combined to use complementary information to enhance the fine-motion classification ability. Transfer learning or domain adaptation methods can be further investigated to reduce the impact of data distribution differences between subjects on model performance. Aiming at the real-time demand of the brain-computer interface, explore model compression and hardware acceleration techniques to improve online classification efficiency. Validate the effectiveness of the model in practical scenarios, such as neurorehabilitation and motor function compensation, and promote deeper integration of theory and application. The framework proposed in this paper provides new ideas for motor imagery EEG classification, and future research will focus on the above directions to deepen the model performance and promote the practical development of brain-computer interface technology.

## Data Availability

Publicly available datasets were analyzed in this study. This data can be found at: BCI Competition IV Dataset 2A dataset.
